# The Prevalence of Oropharyngeal Dysphagia in Acute Geriatric Patients

**DOI:** 10.3390/geriatrics3020015

**Published:** 2018-03-26

**Authors:** Dorte Melgaard, Maria Rodrigo-Domingo, Marianne M. Mørch

**Affiliations:** 1 Center for Clinical Research, North Denmark Regional Hospital, Bispensgade 37, DK-9800 Hjørring, Denmark; 2 Unit of Epidemiology and Biostatistics, Aalborg University Hospital, DK-9000 Aalborg, Denmark; mariarodrigo@rn.dk; 3 Department of Geriatric, North Denmark Hospital, DK-9800 Hjørring, Denmark; m.moerch@rn.dk

**Keywords:** swallowing disorders, elderly, prevalence, mortality, rehospitalization

## Abstract

Oropharyngeal dysphagia (OD) is underdiagnosed and undertreated in many geriatric centers. The aim of this study is to explore the prevalence of OD in acute geriatric patients. The outcome was mortality during hospitalization, mortality, and rehospitalization within 0–30 and 31–180 days of discharge. A total of 313 consecutive acute geriatric patients (44.1% male, mean age 83.1 years (SD 7.8)) hospitalized from 1 March to 31 August 2016 in the North Denmark Regional Hospital were included in this study. The volume-viscosity swallow test and the Minimal Eating Observation Form-II were conducted for each patient in order to screen for OD. A total of 50% patients presented with OD. In the group of patients with OD, significantly more lived in nursing homes; had a lower weight, DEMMI score, and handgrip strength; and smaller circumference of arms and legs compared with non-dysphagia patients. Patients with OD presented an increased length of stay in hospital of one day (*p* = 0.70). Intra-hospital mortality was 5.8% vs. 0.7%, (*p* < 0.001) compared with patients with no symptoms of OD. OD is prevalent in acute geriatric patients, and the mortality is 34% within six months of hospitalization. Screening for OD should be given more attention and included in geriatric guidelines.

## 1. Introduction

It is essential for humans to eat and drink, and dysphagia describes difficulties in meeting these basic needs. There are several definitions of dysphagia, with one being “difficulties moving food from the mouth to the stomach” [1]. The International Classification of Functioning, Disability, and Health (ICF) classifies swallowing as “functions of clearing the food and drink through the oral cavity, pharynx and oesophagus into the stomach at an appropriate rate and speed” [2]. 

Oropharyngeal dysphagia (OD) is highly prevalent in elderly patients in different settings. The prevalence is 30–40% in independently living elderly people [3], and it is reported to be 44–47.4% in patients in acute geriatric units [4,5]. In patients with different forms of dementia, the prevalence is as high as 84% [6,7]. OD may lead to following complications in elderly people: (1) malnutrition and/or dehydration due to lack of efficacy, and (2) choking and aspiration with pneumonia due to lack of safety [8]. Over time, the complications may lead to frailty, social withdrawal, and mortality [5,9,10,11,12,13]. Loss of muscle mass, impaired dental status, reduction of saliva production, and changes in the cervical spine all affect the swallowing function [11]. Older adults are particularly vulnerable to OD due to the fact that disease prevalence increases with age and given age-related changes of the aerodigestive tract, which affects the ability to efficiently and safely swallow [14,15]. Elderly people with OD often experience delayed oropharyngeal swallow response and weak tongue propulsion. This combined with risk factors such as aging, confusion, and medication can lead to impaired safety in swallowing. Weak tongue force related to sarcopenia and reduced bolus prolusion may lead to reduced swallowing efficacy [16]. The European Geriatric Medicine Society and the European Society for Swallowing Disorders have suggested dysphagia to be a geriatric syndrome in line with immobility, instability, incontinence, intellectual impairment, sarcopenia, and frailty. 

In Europe, geriatric patients are not systematically screened for OD, despite the fact that OD is highly prevalent in elderly patients with multifactorial diseases associated with comorbidity and poor outcomes.

The primary aim of this study was to assess the prevalence of OD in Danish patients hospitalized in a geriatric department. The secondary aim was to document rehospitalization and mortality within 30 and 31–180 days of discharge and to document the prevalence of oropharyngeal dysphagia by reason for hospitalization.

## 2. Materials and Methods 

### 2.1. Subjects

**Figure 1:**
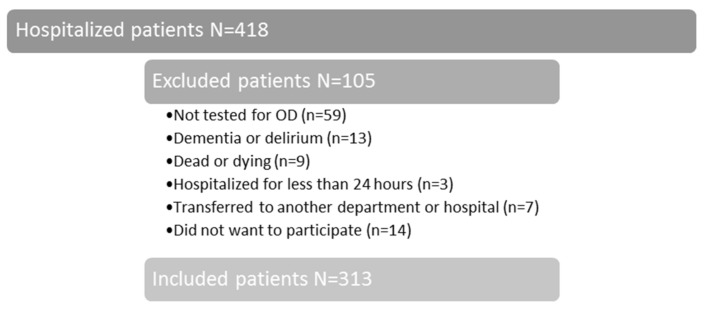
Flowchart over included patients.

A cross-sectional observational study with longitudinal follow-up was applied in the period from 1 March to 31 August 2016. Patients hospitalized in the Department of Geriatric Medicine at the North Denmark Regional Hospital were consecutively screened for OD. Inclusion criteria were ≥60 years old, hospitalized for minimum 24 h and able to cooperate in the test for OD. Of the 418 patients enrolled in the study, 313 (75%) participated. The reasons for exclusion are illustrated in Figure 1. 

The North Denmark Regional Committee on Health Research Ethics (N-20160007) and the Danish Data Protection Authority (2008-58-0028) approved the study.

### 2.2. Procedure and Measurements

Data were collected on body mass index (BMI), waist circumference (2 cm above the navel), strength in the dominant hand, circumference of the lower leg (15 cm above the lower edge of the patella), circumference of the upper arm (lateral epicondyle + 10 cm), Barthel 100, and DEMMI score. Age, gender, admission date, discharge date, Charlson Comorbidity Index (CCI), and primary cause of admission were recorded from the National Patient Register. Rehospitalization was defined as hospitalization due to the disease they were discharged with in the Northern Region of Denmark.

### 2.3. Measurements

OD was screened by using the Volume-Viscosity Swallow Test (V-VST) and the Minimal Eating Observation Form version II (MEOF-II). The V-VST assesses the ability to drink safe and effective. The MEOF-II focus on the ability to eat a meal, including e.g., the sitting position, whether the patient is able to manipulate the food at the plate and to transport the food to the mouth. The complexity of eating and drinking is high and by choosing these two tests, the patient’s ability to eat and drink sufficiently is uncovered clinically. Both tests were administered by trained and experienced occupational therapists. 

The V-VST assesses different types of viscosity and volumes. The bolus volumes were 5, 10, and 20 mL. The bolus viscosity was liquid viscosity (21.61 mPa.s), nectar viscosity (295.02 mPa.s) was achieved by adding 1.2 g of the thickener Resource ThickenUp (Nestlé HealthCare Nutrition) to 100 mL water, and pudding viscosity (3682.21 mPa.s) was achieved by adding 6.0 g of the thickener Resource ThickenUp to 100 mL water. Mineral water at a room temperature of 25 °C was used. Boluses of each volume and viscosity were offered to the patients with a syringe during the test to ensure an accurate measurement of the volume. Before the V-VST, a pulse oximeter was placed on the index finger, and baseline readings were measured before starting the test. During the screening, the following clinical signs of disordered swallowing function were observed: impaired labial seal, oral or pharyngeal residue, and multiple swallows per bolus. According to V-VST, the following clinical signs of impaired safety of swallowing were also observed: changes of voice quality, cough, or decrease in oxygen saturation ≥3% to detect silent aspiration [17]. One or more signs of impaired safety or efficacy indicated OD [18].

The MEOF-II is a measurement tool for assessing elderly patients with OD performance in eating [19]. It consists of nine items in three criteria: (1) Ingestion that includes “manipulation of food on the plate”, “transport of food to the mouth”, and “sitting position”; (2) deglutition includes “ability to chew”, “manipulation of food in the mouth”, and “swallowing”; and (3) energy includes “alertness”, “appetite”, and “eating <3/4 of served food”. A higher score indicates a higher level of dysfunction [20,21]. The patients were observed in a meal consisting of a range of viscosities: e.g., breakfast with yoghurt, bread, apples, coffee, and juice. Patients with a dysfunction in ingestion or deglutition were considered to have OD, but patients with a high score in energy were not because these acute patients may express lower appetite for reasons other than OD. 

The functional level was measured with the de Morton Mobility Index (DEMMI) and Barthel 100 [22,23]: both measurements were developed to measure functionality in elderly people. Comorbidity was measured using the Charlson Comorbidity Index (CCI) [24,25].

### 2.4. Statistical Analysis

Descriptive statistics of the demographic variables include the number and percentage of patients for categorical variables, and median ([5th percentile; 95th percentile]) for continuous variables. Differences between the two study groups were analyzed using Fisher’s exact test or the chi-squared test, as appropriate depending on the number of observations, for categorical variables, and the non-parametric equality of medians test for continuous variables. 

Death status was classified as alive 180 days after discharge (level 1), dead between 31 and 180 days of discharge (level 2), dead within 30 days of discharge (level 3), or in-hospital death (level 4). A trend test was used to determine a possible increasing trend of death status in OD vs. non-OD patients. The follow-up time for rehospitalization was 180 days (6 months) after discharge. The analyses does not include the patients who died during the follow-up period without being rehospitalized. Differences in time to rehospitalization between the OD and non-D groups were analyzed using survival analysis (log-rank test). Throughout the analyses, 95% confidence intervals (CI) were reported, and results with a *p*-value < 0.05 were considered statistically significant. We did not perform any imputation of data. Statistical analyses were performed using Stata Version 14.2 (Stata Corporation, College Station, TX, USA).

## 3. Results

In total, 313 patients were included in the project (44% male, mean age 83.1 years (SD 7.81)). As illustrated in Table 1, 50% of the sample was diagnosed with OD (46% male, mean age 83.6 years (SD 8.15)). Patients with OD had a significantly lower weight (*p* < 0.001), DEMMI score (*p* = 0.001), 30 s. chair stand test (*p* = 0.001), smaller circumference of over arm (*p* = 0.001) and lower leg (*p* = 0.001) and more were living in a nursing home (*p* = 0.004). Patients with OD were discharged after a median of 5 days (2; 14) compared with 4 days (1; 13) (*p* = 0.70) for patients not diagnosed with OD. The difference is not statistically significant, but clinically relevant. 

As illustrated in Table 2, the prevalence of OD is higher among patients hospitalized due to dehydration, fall, or dyspnea. The association between cause of admission and OD is highly significant (*p*-value = 0.009).

The data are presented as number and percentage of patients with OD or not OD for the total of patients hospitalized for each reason.

The mortality in patients with OD is significantly higher than in patients with no OD (trend test *p* = 0.001). Table 3 summarizes the death status and the OD status for all the patients.

The data are presented as number and percentage of patients in the OD and non-OD groups. The *p*-value is from a trend test for association between group and death status.

The difference in readmission up to 180 days after discharge is not significantly different between the OD and non-OD groups (*p* = 0.86). Because less than half of the patients had been readmitted during the follow-up period we cannot report median times to readmission. The 25th percentile for readmission is 30 days (CI 11–49) for the OD group and 36 days (CI 14–50) for the not OD group. 

## 4. Discussion

The present study investigated the prevalence of OD in geriatric patients in order to identify the factors associated with OD and the frequency of rehospitalization and mortality within 30 days of discharge. OD was present in 50% of the patients; the LOS in acute geriatric patients with OD was increased compared with patients with no OD. There was no significant difference between the two groups of patients regarding readmission, but the mortality was significantly higher in patients with OD, which affects the possibility of readmission. Dehydration, fall, and dyspnea as reasons for hospitalization were strongly associated with OD in acute geriatric patients. 

### 4.1. Prevalence

The prevalence of 50% documented in this study further supports the observed prevalence of OD of 44% in an acute geriatric department [4]. Another study documents that the prevalence of OD in patients with delirium and dementia are 59.4% and 73.8%, respectively [26]. In this study, we used the V-VST, it is validated, reliable, and possible to use bedside and in the ward. It is rather fast to use the V-VST and in a daily practice where focus of course is on quality in treatment but also length of stay and time to perform the assessment are very important. The gold standard for assessing OD is video fluoroscopy or fiberoptic endoscopic evaluation of swallowing, as both methods can detect silent aspiration, which is not possible with the bedside test used in this present study [27]. For these reasons, the prevalence of OD is assumed to be underestimated in the present study and other studies where OD is assessed with bedside tests.

### 4.2. Risk Factors

OD is, in other studies, associated with age, functional capacity, frailty, and multi-morbidity [5,28]. In the present study, there was no significant difference between the groups of patients with and without OD according to age and CCI. Unlike other studies, the Barthel 100 score was not significantly lower—although lower in patients with dysphagia [26]. Patients with OD had a significantly lower DEMMI score and more of them were living in nursing homes. Prevalence of OD is higher in patients hospitalized due to dehydration (diminished total body water content [29]), fall, or dyspnea (uncomfortable breathing sensations [30]). The subgroups are relative small and a larger sample size will be relevant to describe the prevalence according to reason of hospitalization. 

Frailty as a term is widely used as a multidimensional syndrome of loss of reserves, such as physical ability, energy, health, and weight loss [31,32] and in relation to acute geriatric patients, it is very relevant. More tools are available to measure frailty, but they are not used routinely in the clinic and studies are showing the clinical value may be weak [33,34]. Nevertheless, it is relevant to compare the prevalence in the present study with primarily frail elderly patients to the group of +65 years able-bodied elderly people where the prevalence is approximately 10% to 30%, but true incidence and prevalence are unknown [35].

### 4.3. Rehospitalization

This present study documents no significant difference between the two groups according to rehospitalization. Some studies document a significantly higher frequency of rehospitalization in patients with OD [26], the lower rehospitalization rate in the present study can be caused by the follow-up in the municipalities. Over 75% of the patients with OD received a rehabilitation plan at discharge and were contacted within an optimal five days after discharge by an OD team consisting of a trained OD therapist (occupational therapist) and a dietitian. Nurses and care assistants in nursing homes are trained in treating patients with OD. This focused intervention may have reduced the risk of rehospitalization. The mortality rate is higher in the group of patients with OD, which of course affects the possibility for readmission.

### 4.4. Mortality

This study confirms the high mortality in patients with OD [10,28]. The 180-days mortality including mortality under hospitalization in this study is 33% and confirms the high mortality reported in other studies. The abovementioned OD team in the municipalities appear to be able to prevent rehospitalization but not mortality.

### 4.5. Strengths and Limitations

A strength in this study is that the screening was performed in 75% of all admitted patients and that the patients were consecutively included. 

A limitation of the study is that patients with severe dementia and delirium were not able to participate and both groups are known to have a relatively high prevalence of OD [6,36]. This may lead to an underestimated prevalence of OD in this study. Data regarding Barthel and BMI are not complete. In observational studies including very frail patients, it may be impossible to collect all information.

Another limitation is that OD was assessed with an at-bedside test. In our clinical setting, it was not possible to assess OD with fiberoptic endoscopic evaluation of swallowing. This would qualify the assessment but more geriatric patients would not be able to cooperate with the assessment and it was not available when this study was performed. We used V-VST, as studies have shown this has a strong correlation with video fluoroscopy [37]. V-VST is considered a useful measurement despite weaknesses concerning using oxygen saturation ≥3% to detect silent aspiration and visualizing pharyngeal residue, and both silent aspiration and pharyngeal residue can only be visualized with an instrumental assessment. V-VST has been recommended in other reviews [38,39], and is translated to Danish, but is not validated in Denmark. MEOF-II is validated and recommended as a measurement for performance of a meal [19]. It is not validated for detecting OD and there is no focus on viscosity of the food, but the OTs performing MEOF-II used food with different viscosities such as bread with toppings, apple, yoghurt, biscuits, and hot and cold fluids in different viscosities.

CCI has been developed and used to measure multimorbidity. In our hospital, it can build on information from the medical records, and it is used in this study. A study from 2012 recommended Cumulative Illness Rating Scale as the most accurate predictor of negative outcome in older people [40]. 

## 5. Conclusions

The prevalence of OD in acute geriatric patients is high (50%). OD in older people causes severe complications with a significant impact on the patients’ health, functionality, and nutritional status. Patients with OD are hospitalized for a longer period and their mortality is higher than in geriatric patients with no OD. 

The results of this study suggest a systematic screening of all acute geriatric patients to optimize the treatment. Further investigation is needed to investigate whether systematic rehabilitation can reduce the frequency of rehospitalization and mortality among acute geriatric patients. 

## Figures and Tables

**Table 1:** Baseline demographics and clinical characteristics between patients with OD vs. patients with no OD. The numbers in parenthesis after the variable name represent the number of patients in each group with available information for that variable. Significant results present an asterisk after the *p*-value.

Variable	OD (N = 156)	Not OD (N = 157)	*p*-Value
Gender, male (156, 157)	72 (46.2%)	66 (42.0%)	0.50
Age, years (156, 157)	84 (69; 95)	83 (71; 94)	0.54
Residence, living in nursing home (156, 157)	28 (18.0%)	9 (5.7%)	<0.01 *
Charlson Comorbidity Index (156, 157)	2 (0; 5)	2 (0; 7)	0.18
Barthel 100 (30, 20)	59.5 (0; 98)	72.5 (9; 95)	0.25
DEMMI (118, 128)	36 (0; 74)	44 (7; 85)	<0.01 *
Weight, kg (81, 97)	62 (42.8; 91.2)	73.7 (47.5; 102.4)	<0.01 *
BMI (80, 93)	23.6 (17.05; 32.9)	27.5 (20.0; 38.1)	<0.01 *
Waist line, cm (84, 111)	96 (73; 125)	105 (82; 126)	<0.01 *
Circumference—upper arm, cm (91, 119)	25.5 (19; 34)	28 (21; 36)	0.01 *
Circumference—lower leg, cm (88, 119)	32 (25; 39)	34 (26; 43)	<0.01 *
Hand grip—dominant hand (88, 119)	18.1 (7; 44.6)	20.9 (8.6; 47.2)	0.047 *
Chair stand test (106, 117)	0 (0; 8)	0 (0; 11)	<0.01 *
LOS in hospital, days (156, 153)	5 (2; 14)	4 (1; 13)	0.70
Rehabilitation plan, made (156, 157)	123 (79%)	107 (68%)	0.03 *

**Table 2:** Prevalence of oral dysphagia by reason for hospitalization.

Reason for Hospitalization	OD	Not OD
All	156 (49.8%)	157 (50.2%)
Pneumonia	11 (52.4%)	10 (47.6%)
Dyspnea	20 (57.1%)	15 (42.9%)
Dehydration	15 (75.0%)	5 (25.0%)
Fall	23 (65.7%)	12 (34.3%)
Reduction in food intake	5 (35.7%)	9 (64.3%)
Infections	31 (47.0%)	35 (53.0%)
Diverse	44 (47.3%)	49 (52.7%)
Pain	7 (24.1%)	22 (75.9%)

**Table 3:** Mortality.

Death Status	OD N = 156	Not OD N = 157	*p*-Value
Survived the first 180 days after discharge	103 (66.0%)	128 (81.5%)	0.001
Died within 180 days of discharge	33 (21.1%)	23 (14.7%)	
Died within 30 days of discharge	11 (7.1%)	5 (3.2%)	
Died in hospital	9 (5.8%)	1 (0.6%)	
